# Galantamine Quantity and Alkaloid Profile in the Bulbs of *Narcissus tazetta* and *daffodil* cultivars (*Amaryllidaceae*) Grown in Israel

**DOI:** 10.3390/metabo11030185

**Published:** 2021-03-21

**Authors:** Dana Atrahimovich, Raviv Harris, Ron Eitan, Menashe Cohen, Soliman Khatib

**Affiliations:** 1 Department of Natural Compounds and Analytical Chemistry, Migal-Galilee Research Institute, Kiryat Shmona 11016, Israel; dana.atr@gmail.com (D.A.); ravivh@migal.org.il (R.H.); 2 Department of Biotechnology, Tel-Hai College, Upper Galilee 1220800, Israel; 3 Northern R&D, Migal-Galilee Research Institute, Kiryat Shmona 11016, Israel; rone@migal.org.il (R.E.); menashec@migal.org.il (M.C.); 4 Analytical Chemistry Laboratory, Tel-Hai College, Upper Galilee 1220800, Israel

**Keywords:** *Amaryllidaceae*, *Narcissus*, alkaloids, galantamine, lycorine

## Abstract

Alkaloids produced by the bulbs of the *Amaryllidaceae* are a source of pharmaceutical compounds. The main alkaloid, galantamine, is a reversible acetylcholinesterase inhibitor and allosteric nicotinic receptor modulator, which slows cognitive and functional decline in mild to moderate dementia due to Alzheimer’s disease. Having a complex stereochemistry, the organic synthesis of galantamine for pharmaceutical uses is highly challenging and not always economically viable, and it is therefore isolated from *Amaryllidaceae* bulbs. In the present study, galantamine was extracted and quantified in *Narcissus* bulbs from five cultivars (cvs.), Fortune, Carlton, Ice Follies, Galilee and Ziva, which were grown in Israel under various conditions. Results show that the cvs. Fortune, Carlton and Ice Follies bulbs contained 285 ± 47, 452 ± 73 and 69 ± 17 µg g^−1^ galantamine, respectively, while the Galilee and Ziva bulbs contained relatively low concentrations of galantamine (1–20 µg g^−1^). Irrigation levels and pruning conditions did not affect the galantamine contents. Additionally, the alkaloids profile of the five cvs. was analyzed and characterized using LC-MS/MS showing that galantamine-type alkaloids were mainly detected in the Fortune and Carlton bulbs, lycorine-type alkaloids were mainly detected at the Galilee and Ziva bulbs and vittatine-type alkaloids were mainly detected in the Ice Follies bulbs. The present research is the first to characterize the alkaloids profile in the *Narcissus* bulbs of Galilee and Ziva, indigenous cvs. grown in Israel. The antiviral and anticancer alkaloids lycorine and lycorinine were the main alkaloids detected in the bulbs of those cultivars.

## 1. Introduction

*Amaryllidaceae* is a family of bulbous flowering plants, characterized by the presence of a biogenetically related group of alkaloids derived from norbelladine. Over 300 different alkaloids have been identified from its species [1]. 

*Narcissus* is a genus of the most important spring-flowering plants of the amaryllis family, *Amaryllidaceae*, widely cultivated worldwide [2]. Physiologically, *Narcissus* species can be divided into two main groups: one that requires low temperatures for flowering and another that does not [3]. Most species belong to the first group, whereas *Narcissus tazetta* cultivars (cvs.) belong to the latter. Two *N. tazetta* cvs., Galilee and Ziva, are characterized by 3–20 flowers per stem and low cold temperature requirements for flowering. Carlton, Fortune and Ice Follies cvs. from the large-cupped division are characterized by a single flower per stem and high cold requirements for flowering [3]. The difference between these two groups is reflected in their popular names. While the large-cupped plants are called *daffodils*, the common name for plants from the *tazetta* group is *Narcissus*. 

**Figure 1:**
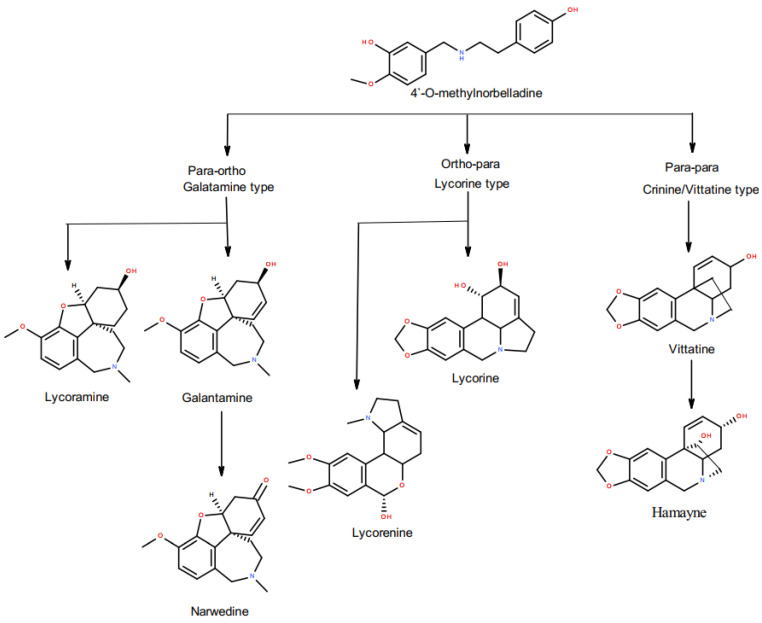
Biosynthetic pathway leading to multiple *Amaryllidaceae* alkaloids.

Galantamine is the most studied alkaloid from *Amaryllidaceae* bulbs [4]. The discovery that it is a reversible inhibitor of acetylcholinesterase, and that it can cross the blood–brain barrier, led to its clinical use for neurological conditions. Galantamine is also recognized as an allosteric modulator of the neuronal nicotinic acetylcholine receptors [5]. It has been shown, both in vitro and in vivo, to have a neuroprotective effect on brain tissues subjected to, for instance, oxidative stress, or oxygen and glucose deprivation [1]. The projected increase in the number of patients with Alzheimer’s disease due to an aging population [6], and the use of galantamine to treat other medical conditions, are likely to result in an increased demand for this compound. *Narcissus* bulbs are considered one of the most important natural sources of galantamine other than synthetic production. Nevertheless, the potential utility of the *Narcissus* species is not just restricted to galantamine, the *Amaryllidaceae* synthesize a diverse array of alkaloids (Figure 1) with pharmacological activities, and compounds like lycorine, lycorinine, homolycorine, haemanthamine, narciclasine and tazettine are known for their anticancer properties [1].

The alkaloid pattern, as well as galantamine content and percentage in the alkaloid mixture, varies widely with geographical location and plant genotype [7]. Obviously, additional growth conditions, such as nutrition, humidity, irrigation, pest control, light levels, pruning and harvest time might affect chemical concentrations and composition. The alkaloid pattern and galantamine contents in the bulbs of the five cultivars of *Narcissus*, Carlton, Fortune, Ice Follies, Galilee and Ziva, grown in Israel under various irrigation levels were characterized. To our knowledge, no investigation has reported on *Amaryllidaceae* alkaloids diversity and accumulation in *Narcissus* bulbs of the Galilee and Ziva cultivars grown in Israel. Hence, our aim was to quantify the galantamine content in the investigated *Narcissus* cvs. bulbs and to analyze their alkaloid profile in order to broaden the variety of cvs. known in the literature and to benefit their pharmacological potential. 

## 2. Results

### 2.1. Extraction and Quantification of Galantamine from Narcissus Bulbs—Method Optimization and Validation 

Three methods were used for the extraction of galantamine from *Narcissus* bulbs. Each of the methods was optimized as described in Materials and Methods and validated by measuring recovery and repeatability. *Narcissus* bulbs were lyophilized, ground and galantamine-d3 was added as the internal standard. Then, the pulverized bulbs were extracted with methanol, and galantamine was further extracted using liquid–liquid extraction (LLE) and solid-phase extraction (SPE) methods. Each extraction method was repeated six times with and without spiked galantamine to calculate the recovery and repeatability. Recoveries of 75%, 64% and 82% were obtained for the LLE, Strata X-C cartridge and Oasis MCX cartridge, respectively. All the extraction methods showed high repeatability (relative standard deviation (RSD) of 2–7%; Table 1). The SPE using MCX cartridges showed the highest recovery, and was used for the extraction and quantification of galantamine in *Narcissus* bulb cultivars.

Galantamine quantification was performed using LC–MS against a standard calibration curve which showed good linearity (r^2^ ≥ 0.999) for a wide range of concentrations, 15 to 5000 ppb (Supplementary Figure S1); the limits of detection (LOD; signal-to-noise (S/N) = 3) value was 0.063 ng mL^−1^ and limits of quantification (LOQ; S/N = 10) value was 0.25 ng mL^−1^.

### 2.2. Galantamine Quantification in Different Narcissus Cultivars Grown under Different Conditions

Five *Narcissus* cultivars were grown under different levels of irrigation and pruning (Table 2). Galantamine concentration in the bulbs was quantified by LC–MS. 

**Figure 2:**
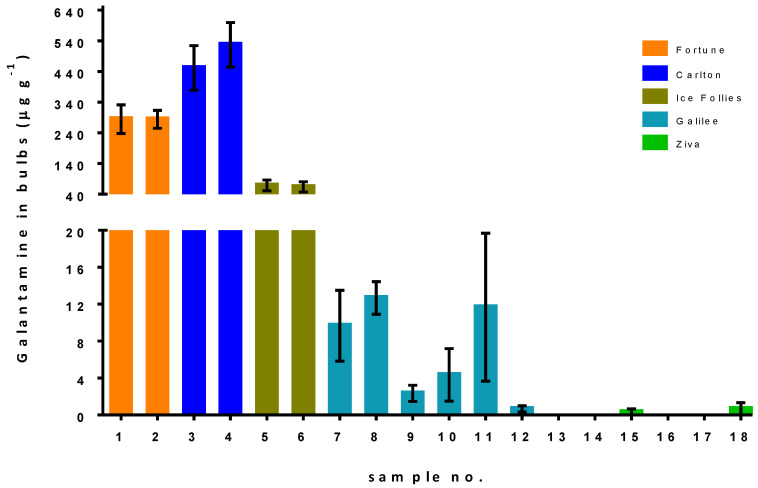
Galantamine concentration in the bulbs of different *Narcissus* cultivars, under different irrigation and pruning levels. Five *Narcissus* cultivars were grown under different irrigation and pruning levels. Sample numbers 1, 3 and 5 represent normal irrigation and samples 2, 4 and 6 represent reduced irrigation. Samples 7, 8 and 9 represent normal irrigation and no pruning, high pruning and low pruning, respectively, with the same conditions for samples 13, 14 and 15. Samples 10, 11 and 12 represent reduced irrigation and no pruning, high pruning and low pruning, respectively, with the same conditions for samples 16, 17 and 18 (see Table 2). The galantamine concentration in the bulbs was quantified using LC–MS/MS. Results are means ± standard error (*n* = 3).

Large differences in galantamine concentration were observed between the cultivars (Figure 2). ‘Fortune’, under normal and reduced irrigation (samples 1 and 2, in Table 2), contained 285 ± 47 µg g^−1^ and 284 ± 29 µg g^−1^ galantamine, respectively; ‘Carlton’ (samples 3 and 4, in Table 2) contained the highest galantamine levels of 452 ± 73 µg g^−1^ and 527 ± 73 µg g^−1^, respectively; ‘Ice Follies’ (samples 5 and 6, in Table 2) contained 69 ± 17 µg g^−1^ and 64 ± 17 µg g^−1^ galantamine, respectively. ‘Galilee’ (samples 7–12, in Table 2) contained relatively low concentrations of galantamine (1–20 µg g^−1^), with no significant effect of the irrigation level or pruning conditions; finally, the galantamine concentrations in the ‘Ziva’ bulbs (samples 13–18, in Table 2) were below the LOQ and were not affected by irrigation or the pruning conditions (Figure 2).

### 2.3. Alkaloid Profiles in Narcissus Cultivars Using LC-MS/MS Analysis

**Figure 3:**
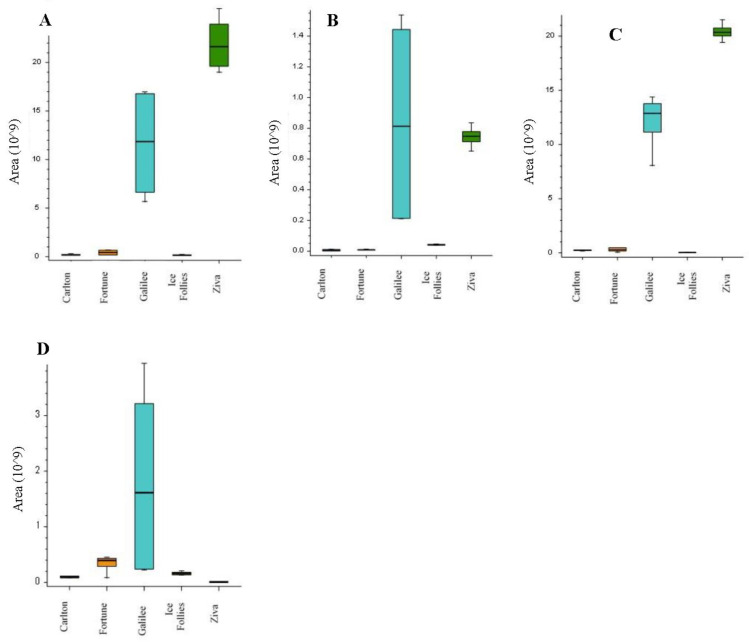
The distribution of the compounds lycorine (**A**), latifaliumin A (**B**), lycorinine (**C**) and tazettine (**D**) detected mainly in the Galilee and Ziva cultivars presented as box plots. The figures present the mean area values obtained from four extracts for each cultivar (*n* = 4), where the limit of the lower and upper quartile and minimum and maximum of the distribution are presented as error bars.

**Figure 4:**
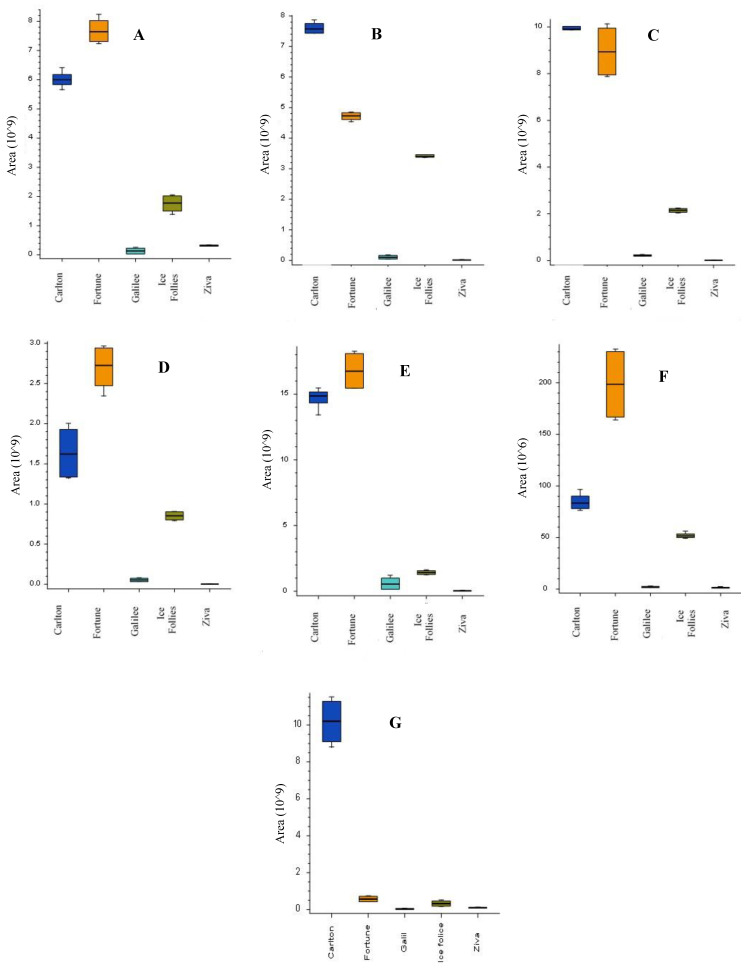
The distribution of the compounds carinatine (**A**), norgalantamine (**B**), galantamine (**C**), lycoramine (**D**), galanthine (**E**), narweden (**F**) and narcissidine (**G**), detected mainly in the Carlton and Fortune cultivars presented as box plots. The figures present the mean area values obtained from four extracts for each cultivar (*n* = 4), where the limit of the lower and upper quartile and the minimum and maximum of the distribution are presented as error bars.

**Figure 5:**
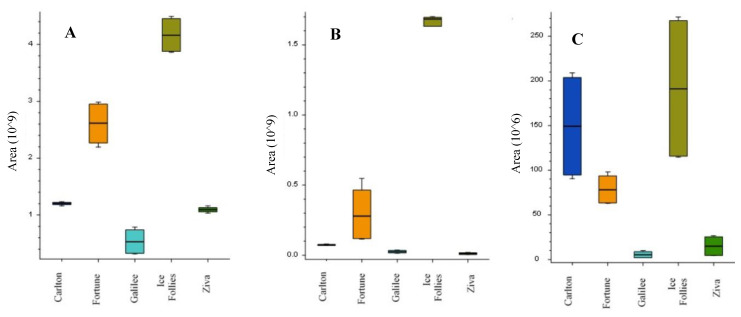
The distribution of the compounds hamayne (**A**), vittatine (**B**) and 4’-O-methylnorbelladine (**C**), detected mainly in Ice Follies cultivar presented as box plots. The figures present the mean area values obtained from four extracts for each cultivar (*n* = 4), the limit of the lower and upper quartile and the minimum and maximum of the distribution are presented as error bars.

The next step was to characterize the alkaloids profile in the bulbs of the five *Narcissus* cultivars. LC-MS/MS analysis using high-resolution MS was performed to the extracts of the five different *Narcissus* cultivars grown under normal irrigation levels without pruning. Four extracts from each cultivar were injected using LC-MS with electrospray ionization plus (ESI+) scan mode. Quality control samples were used for peak area normalization, while only alkaloids with RSD < 15% at the quality control samples were used for analysis. Data-dependent LC-MS/MS analysis was performed for compound identification. Compound Discoverer 3.1 software was used for data processing and analysis. Total ion chromatograms (TIC) obtained from the LC-MS analysis for the five cultivars were presented in the Supplementary Data (Figure S2). Fifteen alkaloids were detected and summarized in Table 3. The molecular formulas of the alkaloids were calculated from the high-resolution exact mass with Δmass < 5ppm. Based on a commercial standard, the compound that appeared at 3.68 min was identified as galantamine. Based on comparisons of MS/MS data with the reported literatures [8,9,10,11,12,13,14], the compounds that appeared at retention time (RT) of 1.75, 2.31, 2.57, 2.99, 3.84, 4.47, 4.89, 5.24 and 5.28 min were identified as latifaliumin C, lycorine, norgalantamine, hamayne, lycoramine, latifaliumin A, galanthine, narwedine and vittatin, respectively (Table 3). Five alkaloids were annotated using Chemspider and MZcloud databases and using a fragment ion search (FISh) scoring algorithm from Thermo Scientific™. FISh scoring uses Mass Frontier™ fragmentation libraries to predict in silico fragments based on the structure of the parent compound. Based on the maximum FISh score coverage, the compounds that appeared at RT of 2.44, 5.24, 5.33, 5.46 and 5.66 min were annotated as carinatine, narcissidine, tazettine, 4’-O-methylnorbelladine and lycorinine, respectively (Table 3 and Supplementary Figure S3). Three main alkaloid clusters were obtained, and each consisted of the further subgroups. The first cluster included four alkaloids, lycorine, latifaliumin A and lycorinine, with greatest abundance mainly in cvs. Ziva and Galilee, and tazettine, with the greatest abundance mainly in Galilee cvs. (Figure 3). The second cluster included carinatine, norgalantamine, galantamine, lycoramine, galanthine, narwedenand and narcissidine, which had greater abundance mainly in the Carlton and Fortune cvs. (Figure 4). The third cluster included the alkaloids hamayne and vittatine, which were mainly abundant in the Ice Follies cvs. (Figure 5). 

In accordance to the results obtained from the galanthamine extraction and quantification section, Figure 4C also presents high levels of galanthamine in the bulbs of Carlton and Fortune cvs., low levels in the Ice Follies cvs. bulbs and extremely low levels in the Galilee and Ziva cvs. bulbs. In contrast, lycorine, which also showed pharmaceutical potential [15], was found at high levels in the bulbs of the Galilee and Ziva cvs., but at low levels in the bulbs of the Carlton, Fortune and Ice Follies cvs (Figure 3A). 

## 3. Discussion

Although galantamine can be chemically synthesized, the associated high costs and a growing demand for this compound have driven scientists to obtain the active ingredient from plant matrices. Thus, plant extracts are still the main production source for this natural product. Generally, sources for galantamine isolation are plants of the *Amaryllidaceae*, usually *Leucojum aestivum* and the *Narcissus* cvs. [7]. Galantamine accumulation is higher in bulbs than in other plant organs, with the exception of the pre-flowering and flower senescence stages [16]. We investigated the alkaloid fraction, with a focus on galantamine content, of five *Narcissus* cvs. belonging to different horticultural and physiological groups. 

The studied *Narcissus* bulbs consisted of two groups: one containing cvs. Galilee and Ziva of the *tazetta* division, and another containing cvs. Carlton, Fortune and Ice Follies of the *daffodil* division. The cvs. were exposed to different irrigation levels (full/reduced) and different pruning conditions during growth (Table 2). At the end of the experiment, the bulbs were collected, counted and divided into three bulb sizes (small, medium, large). Cvs. Galilee and Ziva grew nicely. The propagation coefficient, defined as the number of bulbs yielded per plant, was five, one of which was large. Thus, each planted bulb yielded one alternative bulb for planting in the following year and another four small/medium bulbs that continue to grow for another year and serve as the basis for expanding the growing area. Cvs. Carlton, Fortune and Ice Follies, on the other hand, did not grow well. The propagation coefficient for ‘Fortune’ and ‘Ice Follies’ was one, whereas for ‘Carlton’ it was less than one (0.16). The benefit of reducing the irrigation was pronounced in the *daffodil* group, but the propagation coefficient for large bulbs was low (data not shown). Then, galantamine was extracted and quantified, and the extraction methods were validated by measuring recovery and repeatability (Table 1). Three different extraction methods were used: acid–base LLE and two different types of mixed-mode, strong cation-exchange SPE cartridges. Acid–base LLE is a common method that has been used for the extraction of ionizable compounds for many years, and it is a well-proven technique for the extraction of alkaloids from plant extracts [17]. This method was used as the standard for comparison and demonstrated acceptable recovery of 75% (7% RSD). However, it requires the use of strong and hazardous acids and bases and toxic organic solvents. It is also time-consuming and requires high-precision pipetting to achieve good repeatability. SPE has been an accepted alternative sample preparation method for over 20 years for the analysis of organic compounds [18]. Two different brands of mixed-mode, strong cation-exchange SPE cartridge were tested. Use of the Strata X-C cartridges demonstrated relatively low recovery of galantamine (64%, RSD 2%). However, good recovery of 82% (4% RSD) was obtained with the MCX cartridges. Unlike LLE, the MCX cartridge method requires the use of only mild acids and bases, and methanol is the only organic solvent. The method is also quicker, with better repeatability. An additional advantage of the MCX cartridge method is that galantamine can be extracted directly from the methanolic extract and the entire procedure is water free. 

Overall, the MCX mixed-mode, strong cation-exchange SPE cartridges gave the best results for all of the compared parameters (recovery, speed and environmental and health safety). Therefore, this extraction method was chosen for further quantification of galantamine in *Narcissus* bulbs.

Cvs. Carlton, Fortune and Ice Follies contained relatively high galantamine contents that were not affected by irrigation practice; cvs. Galilee and Ziva, with the lowest galantamine contents (below 20 µg g^−1^), were similarly not affected by the irrigation or pruning conditions (Figure 2). The contents of galantamine can vary widely among *Amaryllidaceae* species, from trace amounts to a few milligrams per gram dry weight (DW) [4]. A wide range of 190 to 990 μg galantamine/g DW was reported for the bulbs of 97 ornamental *Narcissus* varieties [19]. *N. tazetta* populations were found to contain from 30 to 300 μg/g DW galantamine in their bulbs [20]. The galantamine contents of the cvs. in the present study were in line with those reported in the literature.

We further broadened our analysis to the identification of other pharmacologically interesting alkaloid products. Recent studies demonstrated that all the *Amaryllidaceae* alkaloids share a common biochemical pathway with a key intermediate 4′-O-methylnorbelladine, which then undergoes cyclization to give diverse basic skeletons of *Amaryllidaceae* alkaloids (Figure 1) [21,22]. Cyclization of 4’-O-methylnorbelladine can occur by three different ways of intramolecular C–C oxidative phenol coupling, named “para-ortho”, “ortho-para” and “para-para”, that respectively generate three backbone structures. The para-ortho’ C–C coupling leads to galantamine-type alkaloids, the ortho-para’ phenol coupling leads to lycorine-type of alkaloids and the para-para coupling produces the crinine/vittatine and other types of alkaloids (Figure 1) [21,22]. Scanning LC-MS and data-dependent LC/MS/MS were performed to analyze the alkaloids profile of the five *Narcissus* cvs. On the basis of the comparisons of MS/MS data with the reported literatures and FISh scoring algorithm from Thermo Scientific™, 15 alkaloids were identified and annotated respectively (Table 3). Three main alkaloid clusters were obtained. The “ortho-para” was the main C-C cyclization pathway in the Galilee and Ziva bulbs which leads to the synthesis of lycorine-type alkaloids, mainly Lycorine and Lycorinine (Figure 3). The “para-ortho” cyclization was the main pathway in the Fortune and Carlton bulbs, which lead to the synthesis of galantamine-type alkaloids, mainly norgalantamine, galantamine, lycoramine and narweden (Figure 4). The “para-para” cyclization was the main pathway in the Ice Follies bulbs, which lead to the synthesis of vittaine-type alkaloids, mainly Hamayne and Vittatine (Figure 5). 

Although galantamine levels in Galilee and Ziva cvs. were extremely low, the lycorine levels in the Israeli cvs. were the highest. Lycorine has a wide range of biological functions for the treatment of cancer and infectious diseases [23]. Lycorine has been reported to (i) inhibit the export of influenza virus nucleoprotein from the nucleus, and (ii) downregulate autophagy or block the elongation of viral RNA translation during enterovirus 71 (EV71) infection, suppressing viral replication. Altogether, it is speculated that the mechanism behind the anti-SARS-CoV-2 activity of lycorine can be attributed to the modulation of host factors rather than direct targeting of viral factors [24]. 

In conclusion, among the five studied cvs., ‘Galilee’ and ‘Ziva’ grew best with the highest propagation coefficient. Those cvs., however, contained very low galantamine concentrations, as compared to ‘Carlton’ and ‘Fortune’. Galantamine content was not affected by the irrigation or pruning conditions. We further detected another pharmacologically interesting alkaloid in ‘Galilee’ and ‘Ziva’—lycorine, a promising therapeutic agent with antiviral and anticancer properties. 

## 4. Material and Methods

### 4.1. Materials

Galantamine was purchased from Sigma-Aldrich (St. Louis, MO, USA). Galantamine-d_3_ (used as internal standard) was purchased from Cayman Chemical Company (Ann Arbor, MI, USA). All other buffers, solvents and reagents were purchased from Sigma-Aldrich. 

### 4.2. Narcissus Cultivars, Growth Conditions and Bulb Collection

In November 2018, bulbs of three *daffodil* cultivars (Carlton, Fortune and Ice Follies) and two *Narcissus* cultivars (Galilee and Ziva of the *tazetta* division) were planted in the local soil of the Avney Eitan experimental station in the Golan Heights, Israel (375 m above sea level, N 35°45′45″ E 32°02′49″) (Supplementary Figure S4). The two cultivars of the *tazetta* group were divided into six treatments consisting of two levels of irrigation (normal—additional irrigation after cessation of rain in the spring until bulb harvest, and reduced irrigation—without additional watering after cessation of rains), and three levels of simulated grazing (cutting of the top one-third or top two-thirds, or no pruning of the foliage), implemented on 26 February 2019. The three *daffodil* cultivars were tested at the two irrigation levels, for a total of 18 groups in the experiment (Table 2). The bulb crop was harvested on 12 June 2019 and stored in a shaded warehouse at room temperature. 

### 4.3. Extraction of Alkaloids from Narcissus Bulbs

Extraction of alkaloids was performed according to Georgiev et al. [25] with some modifications. *Narcissus* bulbs were lyophilized for 72 h and pulverized. The powder (300 mg) was incubated with 5 mL methanol for 16 h at 37 °C with shaking at 200 rpm. The solution was filtered, and the remaining residue was re-extracted with an additional 5 mL methanol for 30 min; then, the two methanol solutions were combined. A 500-μL aliquot from each sample was evaporated and redissolved with double distilled water (DDW) containing 0.1% formic acid and injected to LC-MS/MS for the analysis of whole alkaloids profile. The remaining crude methanol was further extracted for galantamine quantification. The extraction yields for all the cultivars were 12 ± 3%.

### 4.4. Galantamine Extraction and Quantification Methods

Galantamine-d_3_ was added as an internal standard at a concentration of 1 µg mL^−1^ to the crude extract and then galantamine was extracted using either liquid–liquid extraction (LLE) or cation-exchange solid-phase extraction (SPE) cartridges.

LLE was performed according to the method of Georgiev et al. [25], with some modifications. First, 1 mL of the crude methanol extract solution was transferred into a 4 mL vial and the solvent was evaporated under a nitrogen stream. The residue was dissolved in 1 mL of 3% sulfuric acid (pH = 2.0). Nonpolar compounds were removed by extracting three times with 1 mL diethyl ether for 30 min, followed by the basification of the aqueous fraction (to pH = 11.0) by adding 250 µL ammonium hydroxide. Finally, the galantamine was extracted three times with 1 mL chloroform. The chloroform fractions were combined and evaporated under a nitrogen stream and the residue was redissolved in 2 mL DDW containing 0.1% formic acid (FA). The solution was filtered and injected into an LC–MS instrument for galantamine quantification.

Extraction of galantamine with cation-exchange SPE cartridges was performed with either Oasis MCX cartridges (Waters, Milford, MA, USA), or Strata-X-C cartridges (Phenomenex, Torrance, CA, USA). Each cartridge was conditioned with 1 mL methanol containing 1% FA. The crude extract, obtained from 1 mL methanol, was mixed with 250 µL of methanol containing 1% FA and loaded into the cartridge. The cartridge was then washed with methanol containing 1 mL of 1% FA, followed by a second wash with 1 mL methanol and vacuum-drying. The galantamine was eluted twice with methanol containing 5% ammonium hydroxide. The eluted fraction was evaporated and redissolved in with 2 mL DDW containing 0.1% FA for galantamine quantification by LC–MS/MS.

### 4.5. Galantamine Quantification by UHPLC–HRMS

A 2 μL aliquot of each sample described above was injected into a Dionex Ultimate 3000 ultra-high performance liquid chromatography (UHPLC) system, equipped with a heated electrospray ionization (HESI-II) source connected to a Q Exactive™ Plus Hybrid Quadrupole-Orbitrap™ mass spectrometer (Thermo Fisher Scientific, Bremen, Germany). The extract was analyzed in a Phenomenex 00D-4387-Y0 Synergi Hydro reverse phase liquid chromatography (RP LC ) column (100 × 3 mm I.D., 2.5 µm, 100 Å). The mobile phase consisted of DDW containing 0.1% FA (referred to as A) and acetonitrile containing 0.1% FA (referred to as B). The gradient started with 6% B for 1 min, was increased to 20% B for 2 min, and then increased to 90% B for 2 min and kept at 90% B for another 8 min. Finally, phase B was returned to 6% for 2 min and the column was allowed to equilibrate at 6% B for 3 min before the next injection. The flow rate was 0.5 mL min^−1^. ESI capillary voltage was set to 3500 V, the capillary temperature to 300 °C, the gas temperature to 350 °C and gas flow to 10 mL min^−1^. The mass spectra (*m/z* 100–1000) were acquired using positive ion mode. A calibration curve of galantamine standard was used for galantamine concentration measurement and the same MS parameters were used in positive ion mode. The galantamine concentration was determined using Xcalibur quantification software from Thermo Fisher Scientific, Bremen, Germany.

### 4.6. Galantamine Linearity and Sensitivity

A stock solution of galantamine (100 µg mL^−1^) was prepared in DDW containing 0.1% FA and diluted to obtain a calibration curve ranging from 15 to 5000 ppb (ng/mL). An internal standard was added to each diluted standard (500 ppb final concentration) and used for peak area normalization. Limits of detection (LOD) and quantification (LOQ) were calculated based on a signal-to-noise (S/N) ratio of 3 for LOD and 10 for LOQ.

### 4.7. Validation of Galantamine Extraction Method 

To calculate the galantamine extraction recovery, galantamine–methanol extracts were spiked with a galantamine standard solution (final concentration of 10 µg mL^−1^) before the samples were extracted as described above. In parallel, non-spiked samples were extracted using the same method to determine the initial amount of galantamine present in the sample. The percent recovery was calculated as:

$$\%\;recovery=\;\frac{Galantamine\;in\;spiked\;sample-\;Galantamine\;\;in\;nonspiked\;sample}{Galantamine\;added\;to\;spiked\;sample}\times 100$$

### 4.8. Repeatability

The intra-day repeatability was determined by performing six complete analyses (extraction, sample preparation and quantification) of a representative sample on the same day. The inter-day repeatability was determined by performing the same procedure 3 days later. In between, the samples were stored at −20 °C. The precision of the analytical procedure was expressed as the relative standard deviation (RSD) of the quantified galantamine concentration.

### 4.9. Alkaloids Profile Analysis 

The samples prepared for the alkaloids profile analysis were filtered and injected into the LC–MS/MS as using the same conditions reported above. A pooled matrix was prepared by mixing 20 µL of each experimental sample and used as quality control samples for batch normalization and compound identification. A blank (methanol alone) and quality control sample were injected first in the sequence, after every six samples and at the end of the sequence. The samples were acquired in a scan mode (*m/z* 100–1000) using electrospray-positive ion mode ionization. A data-dependent MS2 analysis was performed for the mixed pool samples and used for compound identification. Peak determination, peak area integration, removal of blank peaks, compound identification using mzCloud and ChemSpider databases, FISh score algorithm and statistical analysis were performed using Compound Discoverer software (Thermo Fischer Scientific, Version 3.1.0.305, Bremen, Germany).

## Figures and Tables

**Table 1:** Recovery and repeatability of extracted galantamine from *Narcissus* bulbs using different extraction methods.

Purification Method	Recovery (%)	RSD (%)
LLE	75 ± 3	7
strata X-C cartridges	64 ± 1	2
mcx cartridges	82 ± 2	4

**Table 2:** Irrigation and pruning levels during *Narcissus* cultivar growth.

Sample No.	Cultivar	Normal Irrigation	Reduced Irrigation	No Pruning	High Pruning	Low Pruning
1	Fortune	V				
2	Fortune		V			
3	Carlton	V				
4	Carlton		V			
5	Ice Follies	V				
6	Ice Follies		V			
7	Galilee	V		V		
8	Galilee	V			V	
9	Galilee	V				V
10	Galilee		V	V		
11	Galilee		V		V	
12	Galilee		V			V
13	Ziva	V		V		
14	Ziva	V			V	
15	Ziva	V				V
16	Ziva		V	V		
17	Ziva		V		V	
18	Ziva		V			V

**Table 3:** The alkaloids detected in the *Narcissus* bulbs of the five cultivars.

Peak Number	Name	Calculated Formula	Molecular Weight	RT [min]	[M+H]^+^	MS/MS	References
1	Latifaliumin C	C_16_H_19_NO_3_	273.1362	1.751	274.144	256.133; 225.091; 217.085; 209.096; 199.075; 184.051	[8,10]
2	Lycorine	C_16_H_17_NO_4_	287.1153	2.31	288.1227	270.1126; 252.102; 240.1057; 177.054; 147.044; 119.049.	[10,14]
3	Carinatine	C_17_H_21_NO_4_	303.1466	2.44	304.1539	286.1434; 268.13297; 193.086; 162.097; 147.044; 119.0495; 95.049; 91.055; 65.039	FISh scoring coverage
4	Norgalantamine	C_16_H_19_NO_3_	273.1361	2.57	274.1435	231.1015; 213.0909; 198.067; 183.044; 169.065; 155.049; 152.062; 115.0546	[10]
5	Hamayne	C_16_H_17_NO_4_	287.1154	2.99	288.1227	270.112; 226.086; 224.07; 211.076; 196.075; 168.0808; 153.069; 152.062; 119.049; 115.054; 91.054; 65.039.	[14]
6	Galantamine	C_17_H_21_NO_3_	287.1517	3.68	288.1233	270.1486; 257.117; 231.101; 225.0908; 213.0909; 198.067; 183.044; 181.065; 169.064; 165.0699; 152.062; 115.055; 91.055.	Standard [10]
7	Lycoramine	C_17_H_23_NO_3_	289.1673	3.84	290.1747	272.164; 233.117; 215.107; 189.091; 145.065; 128.063; 115.055; 95.049; 91.055.	[10]
8	Latifaliumin A	C_16_H_17_NO_4_	287.1153	4.47	288.1591	270.149; 231.102; 225.0914; 213.091; 198.067; 183.044; 169.065; 152.062; 119.049; 115.055; 95.049; 91.055	[8]
9	Galanthine	C_18_H_23_NO_4_	317.1623	4.89	318.1698	300.159; 286.143; 268.133; 193.086; 162.067; 147.044; 119.049; 91.054; 65.039	[14]
10	Narwedine	C_17_H_19_NO_3_	285.1362	5.24	286.1070	257.118; 255.00 229.085; 225.091; 197.096; 181.065; 158.073; 128.062; 115.055.	[10]
11	Narcissidine	C_18_H_23_NO_5_	333.157	5.24	334.1643	316.155; 298.144; 284.126; 266.117; 256.132; 242.117; 165.008; 124.076; 80.05	FISh scoring coverage
12	Vittatin	C_16_H_17_NO_3_	271.1205	5.28	272.128	254.117; 226.086; 224.070; 196.075; 168.08; 139.054; 136.075; 128.062.	[10,14]
13	Tazettine	C_18_H_21_NO_5_	331.1416	5.33	332.1487	300.12296; 282.112; 264.102; 238.086; 225.091; 169.069; 167.085; 165.065; 152.062; 141.069; 139.054; 115.055.	FISh scoring coverage
14	4’-O-methylnorbelladine	C_16_H_19_NO_3_	273.1361	5.46	274.1435	137.059; 122.037; 94.042; 66.039; 65.039.	FISh scoring coverage
15	Lycorinine	C_18_H_23_NO_4_	317.1624	5.66	318.1697	300.159; 286.144; 268.133; 257.118; 258.148; 224.07; 175.075; 147.044.	FISh scoring coverage

## Data Availability

The data presented in this study are available on request from the corresponding author.

## Supplementary Materials

The following are available online at https://www.mdpi.com/2218-1989/11/3/185/s1; Figure S1: (A) LC-MS chromatogram of galantamine and (B) calibration curve of galantamine; Figure S2: Total ion chromatograms (TIC) for the five cultivars Fortune, Carlton, Ice Follies, Galilee and Ziva (black, red, green, blue and yellow chromatograms, respectively); Figure S3: MS/MS spectra and FISh scoring coverage for the annotated alkaloids carinatine (A), narcissidine (B), tazettine (C), 4’-O-methylnorbelladine (D) and lycorinine (E); Figure S4: Map of the *Narcissus* (*Amaryllidaceae*) cultivars planted site in the local soil of the Avney Eitan experimental station in the Golan Heights, Israel (375 m above sea level, N 35°45′45″ E 32°02′49″).

## Abbreviations

Cvs., Cultivars; *N. tazetta*, *Narcissus tazetta*; LLE, liquid–liquid extraction; SPE, solid-phase extraction; LOD, Limits of detection; LOQ, Limits of quantification; S/N, signal-to-noise; RSD, relative standard deviation; DW, dry weight; HESI-II, heated electrospray ionization; EV, enterovirus.
