# Supplementary material for: Galantamine Quantity and Alkaloid Profile in the Bulbs of Narcissus tazetta and daffodil cultivars (Amaryllidaceae) Grown in Israel
Source: Metabolites. 2021 Mar 21;11(3):185. doi: 10.3390/metabo11030185 (PMC8004262; doi:10.3390/metabo11030185)
Supplement: Supplementary file 1 [file metabolites-11-00185-s001.pdf]

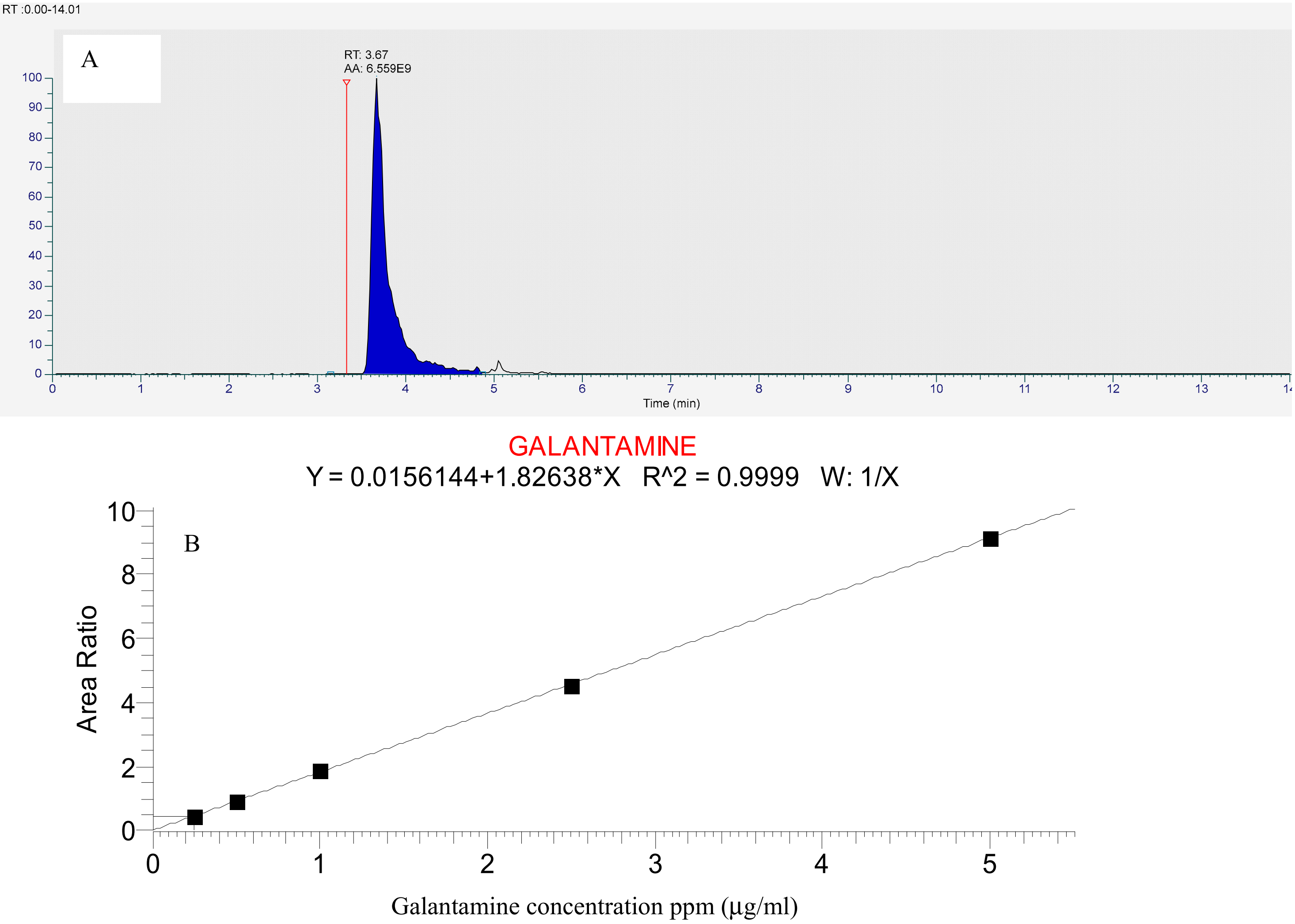

Figure S1: A) LC-MS chromatogram of Galantamine and B) calibration curve of Galantamine.

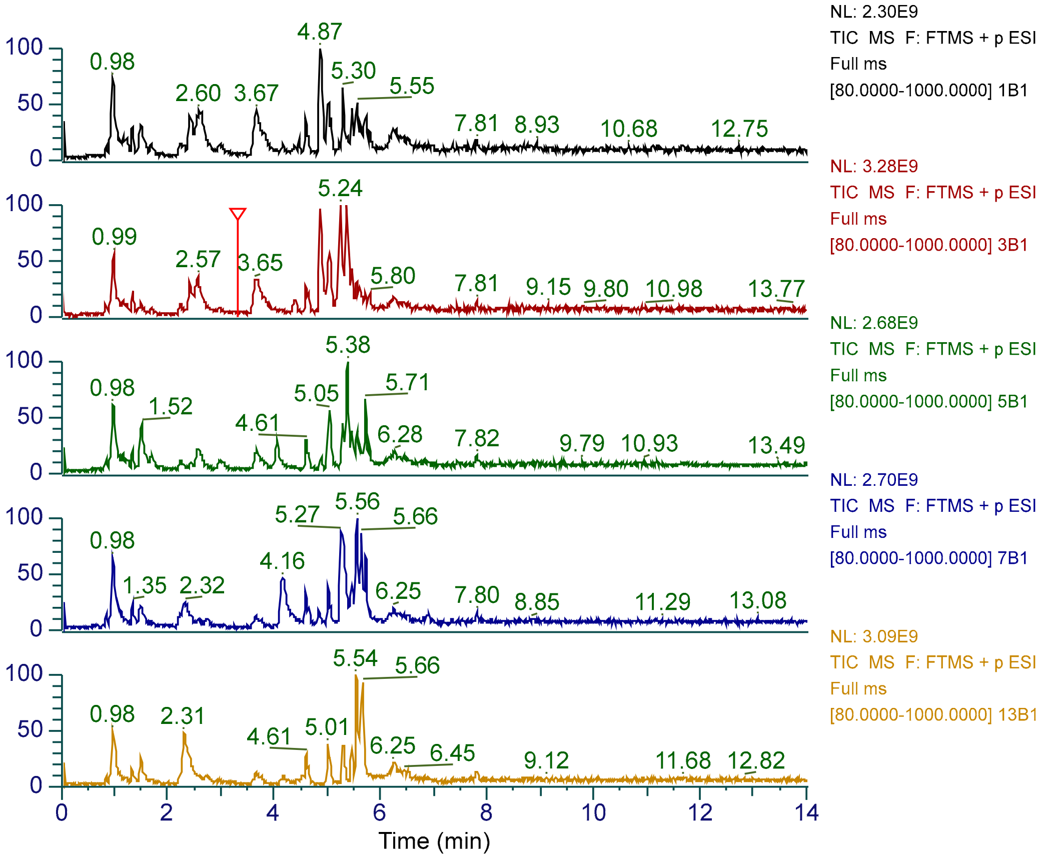

Figure S2: Total ion chromatograms (TIC) for the 5 cultivars Fortune, Carlton, Ice folce, Galil and Ziva (Black, Red, Green, Blue and Yellow chromatograms respectively). The chromatograms obtained from HR LC-MS analysis using ESI^+ scan mode (m/z=80-1000).

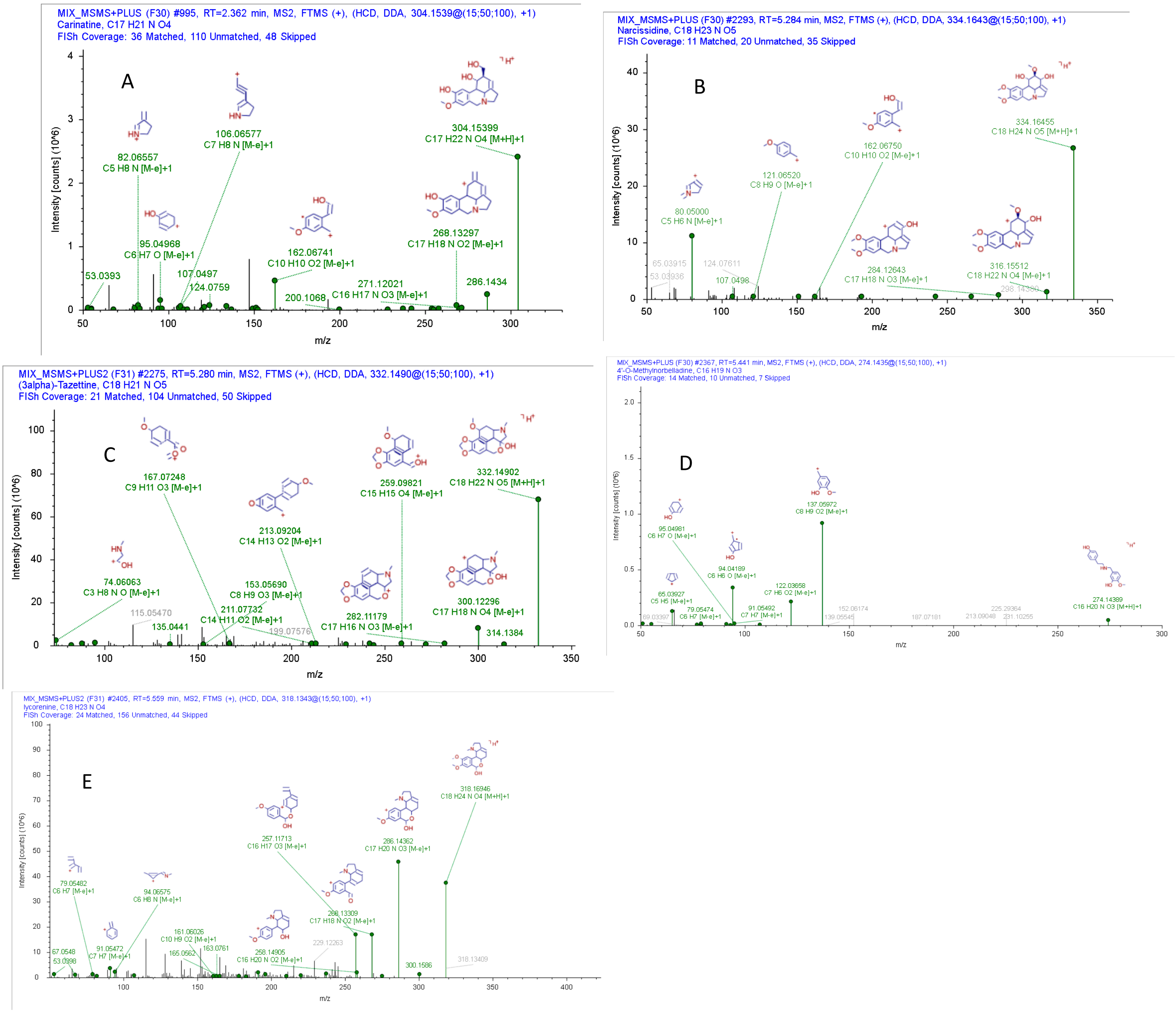

Figure S3: MS/MS spectra and FISh scoring coverage for the annotated alkaloids Carinatine (A), Narcissidine (B), Tazettine (C), 4`-O-methylnorbelladine (D) and Lycorinine (E).

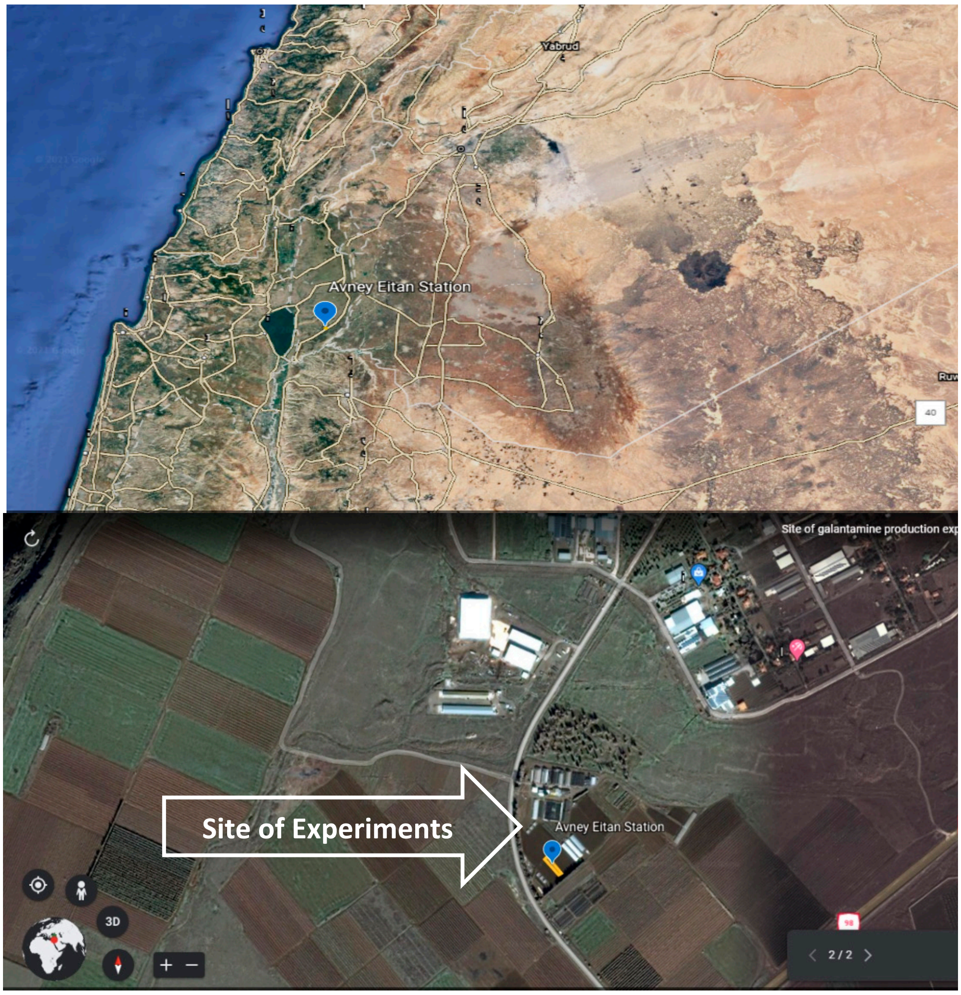

Figure S4: Map of the *Narcissus* (*Amaryllidaceae*) cultivars planted site (In the local soil of the Avney Eitan experimental station in the Golan Heights, Israel (375 Meters Above Sea Level, N35o45'45" E32°02'49"). The maps were taken from Google earth.
